# Coronavirus disease 2019 communication: novel sign language system to aid surgical tracheostomy whilst wearing a respirator

**DOI:** 10.1017/S0022215120001255

**Published:** 2020-06-18

**Authors:** S Wilkinson, E Irvine, T Valsamakis

**Affiliations:** ENT Department, Leicester Royal Infirmary, UK

**Keywords:** COVID-19, Tracheostomy, Sign Language, Respirators, Communication

## Abstract

**Background:**

The coronavirus disease 2019 pandemic has necessitated rapid adaptations to all levels of clinical practice. Recently produced guidelines have suggested additional considerations for tracheostomy and advocated full personal protective equipment, including filtering facepiece code 3 masks. Air seal with filtering facepiece code 3 masks is often challenging, and full-face respirators and powered air-purifying respirators with hoods need to be employed. The infection prevention benefits of this equipment are accompanied by potential issues in communication.

**Objective:**

In an attempt to minimise surgical error through miscommunication, the authors sought to introduce a simple sign language system that could be used as an adjunct during surgery.

**Results:**

Following evaluation of pre-existing sign language platforms and consideration of multiple surgical factors, 14 bespoke hand signals were ultimately proposed.

**Conclusion:**

Whilst this novel sign language system aims to bridge the communicative gap created by additional personal protective equipment, further development and validation of the proposed tool might be beneficial.

## Introduction

Surgical tracheostomy is a common surgical procedure, most frequently performed by otorhinolaryngologists. In 2015, Rangasami and Higgs reported that 1200 tracheostomies were performed annually in the UK, of which approximately 30 per cent were conducted surgically.^1^ Under normal circumstances, tracheostomy is performed using well-established surgical practices. The recent coronavirus disease 2019 (Covid-19) pandemic, however, has necessitated rapid adaptations to a number of long-standing surgical techniques, with tracheostomy receiving particular interest because of the aerosol generating nature of the procedure.

In March, ENT UK published detailed guidance on additional precautions and considerations for tracheostomy throughout the Covid-19 pandemic.^2^ In particular, alterations to endotracheal tube positioning during formation of the tracheal window and the cessation of ventilation with an open airway were advised. In addition, adequate personal protective equipment (PPE) in the form of gloves, fluid-resistant surgical gowns, filtering facepiece code 3 (FFP3) masks and full-face visors was strongly advocated.

International shortages in appropriate protective equipment for medical professionals during the current pandemic has been widely publicised in the medical literature.^3^ In the USA, a recent national survey identified that up to 91 per cent of cities were experiencing inadequate supplies of face masks, with 88 per cent also reporting insufficient PPE for frontline workers and first responders.^4^ Reduced access to FFP3 face masks, especially at the early stages of the pandemic, as well as issues in air seal during mask fit testing,^5^ has resulted in many UK hospital trusts seeking alternative means of protecting its workforce. Locally, this has been achieved through the acquisition of full-face respirators (PureFlow^™^ PF1000) and Dräger^™^ powered air-purifying respirator hoods.

Despite the obvious infection prevention benefits achieved through the utilisation of such equipment, early clinical experience in our institution has identified a number of issues pertaining to communication whilst fully donned. Interestingly, similar challenges have also been noted within the aviation industry. In 2011, Thomas *et al*. conducted a study comparing the accuracy of transmitted aviation terms using a variety of surgical facemasks and N95 respirators.^6^ Whilst radio reception accuracy was generally high with a surgical facemask, use of a respirator or the addition of background noise appeared to adversely affect the reliability of communication.

In an attempt to minimise surgical error through miscommunication, we sought to develop a simple sign language tool to act as a useful adjunct during surgical tracheostomy.

## Tool design and development

As part of the design and development process, a number of different factors were considered. Each are discussed below, following broadly the headings of: surgical tracheostomy (key operative stages); commonly utilised surgical instruments; and pre-existing sign language frameworks. In a bid to increase usability, attempts were made to minimise the number of hand signals created, whilst still ensuring that the fundamental stages of the procedure were covered.

### Tracheostomy stages

Authors’ practice and the recently published ENT UK guidelines on surgical tracheostomy were reviewed prior to deciding upon the key surgical stages to be incorporated within the tool. Particular attention was paid to the newly suggested steps aimed at minimising the aerosolisation of viral droplets and transmission to healthcare professionals. Table 1 outlines the main operative stages considered during the design process. Additional, non-surgical aspects of the procedure, such as confirmation of an intact tracheostomy tube balloon, were also deemed vital and therefore included.

**Table 1.** Key operative stages of tracheostomy

Key operative stages
Initial incision (performed with number 10 blade)
Division & management of thyroid isthmus
Tracheostomy tube check (including balloon)
Advancement of endotracheal tube
Cessation of ventilation
Formation of tracheal window (performed with number 11 blade)
Insertion of tracheostomy tube (including cuff inflation)
Confirmation of carbon dioxide

Following the compilation of the above list, the management and division of the thyroid isthmus was discounted from the final sign language system. It was the authors’ belief that development of distinct hand signals for each of the potential approaches to the thyroid isthmus would result in too complex an array of hand signals and detract from the overall aim of the tool.

### Commonly utilised instruments

As part of this process, commonly used surgical instruments, as well as those confined solely to surgical tracheostomy were considered (Table 2). Those not utilised during key stages of the procedure or equlpment readily available to the surgical team (e.g. suction and bipolar) were subsequently discounted to minimise confusion.

**Table 2.** Instruments considered in the communication tool design process

Surgical instruments
Scalpel (number 10 & 11 blades)
Bipolar diathermy (pre-set to 12)
Suction
Tracheal dilators
Cricoid hook
Sutures (silk + Vicryl®)
Tracheostomy tube

Additional consideration and clarification for instruments requiring variable sizes or attachments (e.g. sutures, scalpel blades and tracheostomy tube size) was required. In an attempt to overcome this issue, the authors proposed a multi-stage hand signal in which the size of instrument or adjunct required preceded that of the instrument itself. In order to further simplify communication during tracheostomy, it was the authors’ suggestion that settings for equipment utilised during the operation, as well as the appropriate tracheostomy tube size along with a sequence of alternative sizes, be arranged prior to the donning of PPE (e.g. diathermy power).

### Review of pre-existing sign language frameworks

During the design of the communication tool, many pre-existing sign language frameworks were reviewed. These included the British Sign Language system,^7^ as well as hand signals adopted by the military^8^ and scuba diving community.^9^ Where appropriate, transferrable hand signals were incorporated into our tracheostomy specific tool. For example, the universally accepted signal for ‘OK’, in which the thumb and index fingers are opposed to form a loop whilst the third, fourth and fifth digits are extended, was deemed an appropriate signal to be used when clarifying correct tube placement with carbon dioxide. In addition, simple hand gestures to denote letters of the alphabet (British Sign Language) were adopted by the authors as a useful technique to convey the specific object required when dealing with more than one possible item (e.g. suture type). Signals requiring the hands to be in close proximity to the face or mouth were subsequently discounted given the potential risk of de-sterilisation.

### Finalised hand signals

**Fig. 1.**
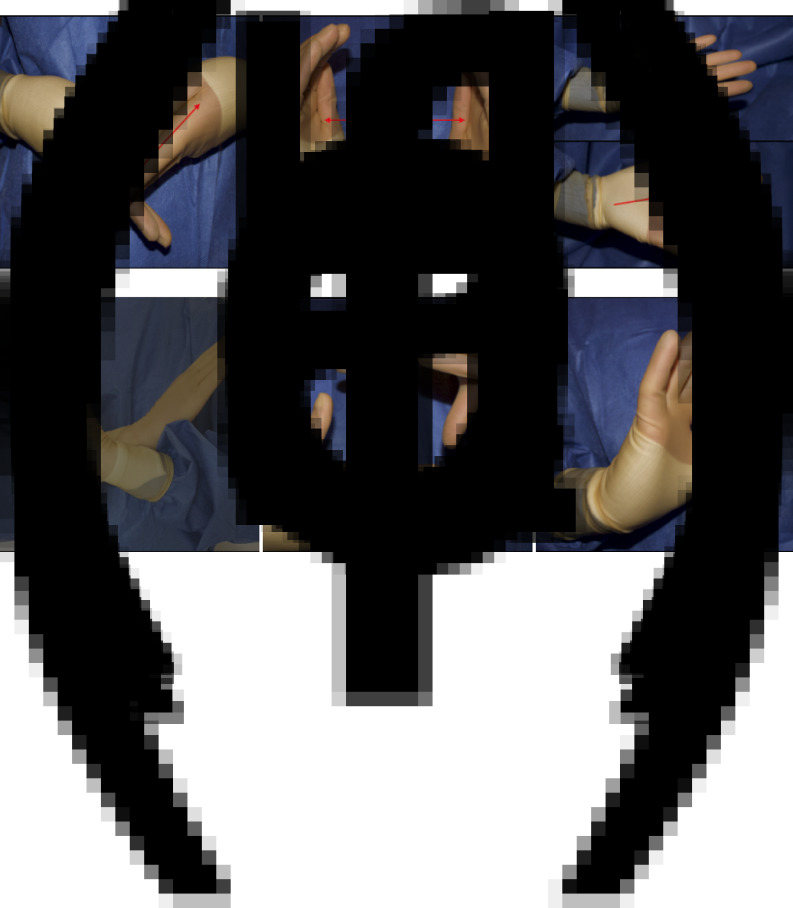
Proposed hand signals for each tracheostomy stage: (a) knife or scalpel, (b) check balloon, and inflate or deflate cuff, (c) advance endotracheal tube, (d) cease ventilation, (e) form tracheal window, and (f) confirm tube position (arrows indicate hand movements)

**Fig. 2.**
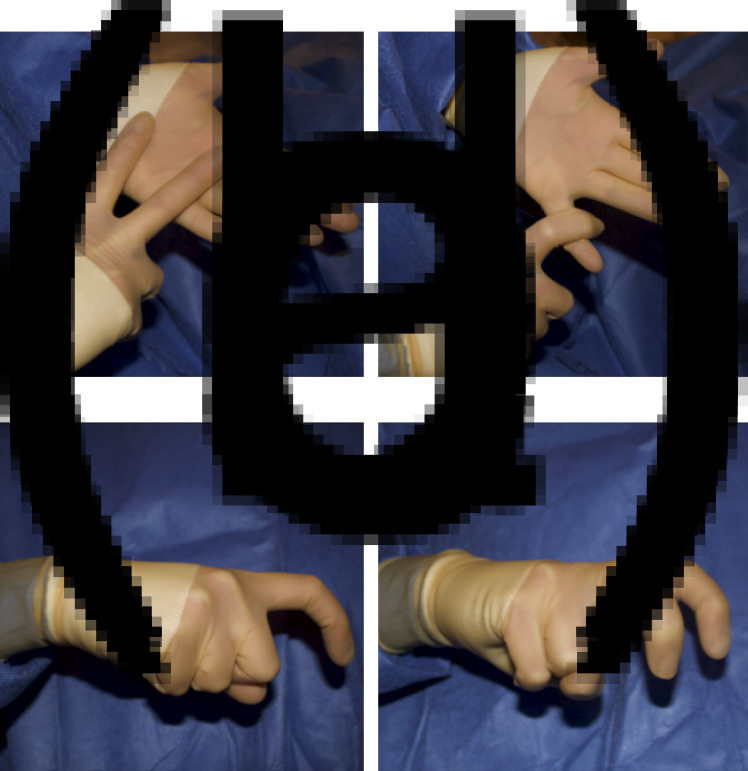
Proposed hand signals for key instruments required during tracheostomy: (a) vicryl, (b) silk, (c) cricoid hook and (d) tracheal dilators.

**Fig. 3.**
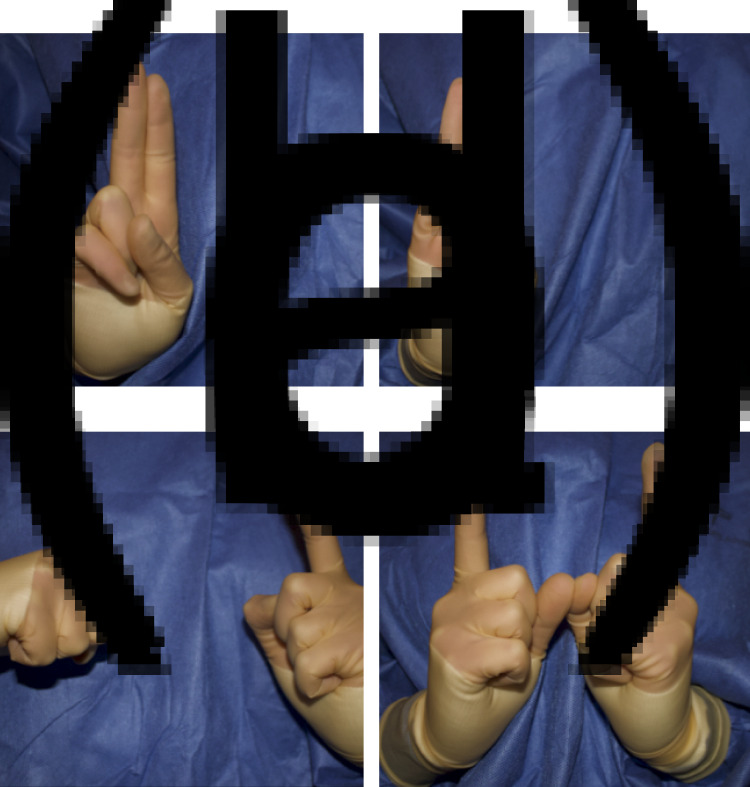
Proposed hand signals for clarification of instrument size or type by surgical team: (a) 2, (b) 3, (c) 10 and (d) 11.

The finalised hand signals are shown in Figures 1–3.

## Discussion

Information transfer and communication during surgery is vital to ensure patient safety and optimise overall outcomes.^10^ In addition to direct instruction through verbal communication, more subtle non-verbal cues in the form of touch or altered body position are thought to play an important role in the operating theatre.^11^ However, recent events (Covid-19) have created surgical environments in which traditional communication techniques are considerably impaired.

Literature pertaining to the impact of respirators on communication within the medical field is far from plentiful. Published data suggest reduced levels of speech intelligibility, to varying statistical degrees, whilst wearing certain models of respirators.^12^ The level of intelligibility has also been shown to decrease when communication is solely telephone dependent.^13^

## Conclusion

To the authors’ knowledge, there has been no published research detailing the use of sign language as an adjunct to communication within the operating theatre. This proposed novel sign language system attempts to bridge the communication gap created with the use of additional PPE, and in doing so minimise errors that may occur as a result of this unfamiliar, sensory-impaired environment.

Whilst the proposed sign language system acts as a good initial reference tool, it is important to note that local adaptation may be required dependent on the surgeon's technique and preference, and the operating theatre team practice. Further development and validation of the above proposed tool might be beneficial in the current era.
